# Human-Derived A/Guangdong/Th005/2017 (H7N9) Exhibits Extremely High Replication in the Lungs of Ferrets and Is Highly Pathogenic in Chickens

**DOI:** 10.3390/v11060494

**Published:** 2019-05-29

**Authors:** Shuran Gong, Feifei Qi, Fengdi Li, Qi Lv, Guanpeng Wang, Shunyi Wang, Jing Jiang, Lin Wang, Linlin Bao, Chuan Qin

**Affiliations:** 1 Institute of Laboratory Animal Sciences, Chinese Academy of Medical Sciences (CAMS) & Comparative Medicine Center, Peking Union Medical Collage (PUMC), 100021 Beijing, China; gongshuran@cnilas.org (S.G.); qifeifei@cnilas.org (F.Q.); icyli1019@hotmail.com (F.L.); lvqi@cnilas.org (Q.L.); wangguanpeng@cnilas.org (G.W.); wangshunyi@cnilas.org (S.W.); jing_jing_jiang@163.com (J.J.); wanglin@cnilas.org (L.W.); 2 Key Laboratory of Human Disease Comparative Medicine, Ministry of Health, Beijing Key Laboratory for Animal Models of Emerging and Reemerging Infectious, 100021 Beijing, China

**Keywords:** H7N9, ferret, mouse, chicken, pathogenicity, transmissibility

## Abstract

After a series of studies on the pathogenicity of several H7N9 strains from 2013 to 2018, we wanted to dynamically track the pathogenicity of A/Guangdong/Th005/2017 in ferrets and poultry. The pathogenicity and transmissibility of Th005, especially the distribution and replication in tissues, were studied in ferrets. We also aimed to assess the level of Th005 pathogenicity in chickens. The results showed that the pathogenicity of Th005 was significantly increased in ferrets and chickens, especially compared with the Anhui strain. The replication of Th005 in the lung tissues of ferrets was 100-fold higher than that of the Anhui strain. Th005 pathogenicity reached an intravenous pathogenicity index (IVPI) score of 3 in avian models. Continuously high titres of viruses could be detected in the cloacal cavity of chickens infected with Th005. Th005 remained highly pathogenic in mice and chickens after passaging in ferrets. High expression of both the α2,6- and α2,3-sialic acid residues in cells in vitro was beneficial to Th005 replication, which was enhanced compared to the Anhui strain. China needs to strengthen its surveillance of virulent influenza virus strains, such as Th005, which continues to increase in pathogenicity.

## 1. Introduction

The first human infection with the influenza H7N9 strain appeared in the Yangtze River Delta region in March 2013 [1]. Between January 2013 and January 2019, the virus caused 1567 infections and 615 deaths. It is worth noting that the number of cases that occurred during the fifth H7N9 wave was equal to the sum of the previous four waves [2]. In addition, molecular biological analyses have shown that the H7N9 influenza virus has changed from low pathogenicity to high pathogenicity [3]. Furthermore, the world organization for animal health (OIE) has reported numerous chicken deaths in poultry farms in China, with H7N9 avian influenza viruses isolated from chickens [4]. It is unclear how the H7N9 viruses have spread in nature and become highly pathogenic. Therefore, further investigation is required.

We collected the Anhui strain from the first H7N9 outbreak in 2013 and thoroughly analyzed the pathogenicity and transmissibility of this H7N9 virus in ferrets and mice. Afterward, our research team collected H7N9 influenza viruses isolated from the fifth wave of the outbreak and carried out a series of tracking and comparison analyses. In the fifth wave of the H7N9 epidemic, the number of patients surged, and the intervals from illness onset to diagnosis and then to death were shorter when compared with the previous lowly, pathogenic avian influenza virus (LPAIV; H7N9)-infected patients. At the same time, the case fatality rate increased from 30% to 40% at the fifth wave [5,6,7,8,9].

The purpose of tracing the variation trends of H7N9 influenza viruses in nature is to dynamically study the changes in the pathogenicity of these viruses and to analyze the correlation between these changes and the aggravation of clinical symptoms. Importantly, we did not genetically modify the virus during this process. To solve the above problems, we evaluated the changes in the pathogenicity of the human-derived Th005 virus in ferret and avian models and determined whether the patient-isolated viruses had higher pathogenicity in poultry, as measured by the intravenous pathogenicity index (IVPI). An attempt was made to analyze the source of the high pathogenicity of the Th005 virus in ferrets and poultry especially the pathogenic changes of the human-derived Th005 after its passage in ferrets and the amount of viral shedding after passaging in chickens, so as to evaluate the hazard level of this virus in nature. These studies provide a foundation for updating risk assessment and developing more appropriate market monitoring measures in the future.

## 2. Materials and Methods

### 2.1. Ethics and Biosafety Statements

The Institute of Animal Care and Use Committee of the Institute of Laboratory Animal Science at the Peking Union Medical College evaluated and approved the animal experimental protocols on 15 March 2017 (#BLL17005). All animals were allocated randomly to experimental groups. All experiments were performed under ABSL-3 conditions.

### 2.2. Viruses

The H7N9 strains A/Guangdong/Th005/2017 (abbreviated Th005) [7] and A/Anhui/1/2013 (abbreviated Anhui) [10] were isolated from the fifth and first infectious waves, respectively. The ferret-passaged Th005 or ferret-passaged Anhui strains were obtained through intranasal inoculation of the primary viruses, Th005 or Anhui, into ferrets. Viruses were obtained 5 days post-infection (d.p.i.) from nasal swab supernatants and lung tissue homogenates and were propagated in embryonated chicken eggs. The Th005 chicken-passaged strain was obtained through intravenous inoculation of the primary virus into chickens. Viruses were obtained at the time of chicken death from nasal swab supernatants and lung tissue homogenates and were propagated in embryonated chicken eggs. The chicken-passaged Anhui strain was obtained via intravenous inoculation of the primary virus into chickens. Viruses were collected at 5 d.p.i. from nasal swab supernatants and propagated in chicken embryos. Each passage strain was obtained by one passage. (The Th005 ferret- and chicken-passaged strains are abbreviated as Th005 FR and Th005 CK, respectively. The Anhui ferret-passaged and chicken-passaged strains are abbreviated as Anhui FR and Anhui CK, respectively.).

### 2.3. Cells

MDCK, A549, and Calu-3 cells were obtained from the American Type Culture Collection and tested for mycoplasma contamination. The cells were maintained in Eagle’s Minimal Essential Medium (MEM, Gibco, Grand Island, NY, USA) supplemented with 10% foetal bovine serum (FBS), 100 IU/mL penicillin, and 100 μg/mL streptomycin and cultured at 37 °C with 5% CO_2_.

### 2.4. Growth Kinetics of H7N9 Viruses in Cells

Cells were cultured in MEM containing 10% FBS. A viral solution diluted to 100 TCID_50_ (median tissue culture infective dose) was inoculated into 35-mm culture dishes containing cell monolayers. After incubation for 1 h at 37 °C, the diluted viral solution was replaced with 3 mL of serum-free culture medium. Cell supernatants were collected at 0, 12, 24, 36, 48, 60, 72, and 96 h post-infection and real-time PCR determined the RNA copy number of the viruses.

### 2.5. Viral Receptor-Binding Characteristics

Chicken red blood cells (CRBC, Beijing Farm Animal Research Center, Institute of Laboratory Animal Sciences, Chinese Academy of Medical Sciences) and α-2,3-sialidase (50 mU/μL, TaKaRa, Shiga, Japan) were mixed at a ratio of 9:1 and incubated at 37 °C for 10 min. After two washes with PBS, the solution was resuspended to create a 0.75% red blood cell (RBC) suspension. Next, 100 µL of 10% CRBC was mixed with 50 mU of *Vibrio cholerae* neuraminidase (VCNA, Sigma-Aldrich, St. Louis, MO, USA), incubated at 37 °C for 1 h, washed 3 times with PBS, and then mixed with 50 µL of PBS. CRBCs and sheep RBCs (TaKaRa, Japan) were prepared as 0.75% RBC suspensions to assess the agglutination characteristics of Th005 and Anhui. The agglutination characteristics of the human influenza virus CA/07 (H1N1) and the avian influenza virus AH/05 (H5N1) with known receptor types were also assessed and used as controls.

### 2.6. Ferret Experiments

Adult castrated ferrets (*Mustela putorius furo*) aged 6 to 10 months were obtained from the Institute of Laboratory Animal Sciences at the Chinese Academy of Medical Sciences and confirmed to be serologically negative (anti-H1, H3, H5, and H7). For acclimatization and reducing the stress response of the animals, all ferrets were implanted with a microchip subcutaneously and entered the ABSL-3 laboratory in advance for 7 days before the infection (body temperature was monitored by microchip scanner, BMDS, Delaware, DE, USA). To study the pathogenicity of these viruses in mammals, three ferrets in the infection group were intranasally inoculated with virus at 10^7^ TCID_50_. After five days, two ferrets in the infection group were randomly selected for euthanasia to collect nasal turbinates, tracheas, lungs, livers, spleens, kidneys, brains, intestines, and eyes for viral titre determination and pathological detection.

To study the aerosol transmissibility of viruses between ferrets, a transmission group was placed next to the infection group, with three ferrets each in the infection and transmission groups. The transmission group was housed in a specialized transmission cage [10] and the infection group was housed in a cage adjacent to the transmission group 24 h after infection. The ferrets in the infection and transmission groups were placed 8 cm apart with a double-layer net between them, such that they could not contact each other, but that air flow could pass freely. The air flowed from the infection group to the transmission group with an air flow velocity of 0.1 m/s.

The body temperatures, body weights, and clinical symptoms of the ferrets in the infection and transmission groups were continuously measured from 0 to 14 days post-infection/exposure. The overall clinical score was divided into two parts, as described by a previous study [11]. Nasal swabs were collected from both groups on 0–7, 9, 11, and 14 days after infection/exposure for viral titer determinations. Blood samples were collected on day 21 for determination of hemagglutination inhibition (HI).

### 2.7. Chicken Experiments

The Th005 and Anhui strains were each used to infect ten SPF-grade, white, six-week-old Leghorn chickens (Boehringer Ingelheim Vital Biotechnology, Beijing, China). Each chicken was intravenously inoculated with 0.1 mL of a 1:10 dilution of bacterium-free allantoic fluid containing the virus. After infection, clinical symptoms and death were continuously recorded for 10 days. The IVPI was determined according to the standard program of the OIE [12]. The reported IVPI was the average score of the clinical symptoms of each chicken observed over 10 days. Death of an animal within 24 h was assigned 3 points, and an absence of symptoms in animals by 10 days was assigned 0 points. Scores of 1.3 points or above indicated that the virus was highly pathogenic. For the symptom scores, 0 points represented normal health and 1, 2, and 3 points indicated sick, severely sick, and dead animals, respectively. The presence of one symptom was regarded as “sick,” and the presence of two or more symptoms was regarded as “severely sick.” The symptoms included respiratory system-related symptoms, depression, diarrhea, cyanosis of the comb, edema of the face, and nervous system symptoms.

The Th005, Th005 FR, Th005 CK, Anhui, Anhui FR, and Anhui CK strains were individually used to infect five six-week-old, SPF-grade, white Leghorn chickens. The intranasal inoculation dose per chicken was 10^6^ TCID_50_. Body weight loss, mortality, clinical symptoms, and rectal temperatures were measured from 0 to 14 d.p.i. Oropharyngeal and cloacal swabs were collected on 0–7, 9, 11, and 14 days for viral titer determination.

### 2.8. Mouse Experiments

The Th005, Th005 FR, Th005 CK, Anhui, Anhui FR, and Anhui CK strains were each used to infect twenty 4-week-old to 6-week-old, female BALB/c mice (Vital River Laboratories, Beijing, China). Each mouse was intranasally inoculated with 0.05 mL of diluted viral solution at a concentration of 10^5^ TCID_50_/mL. Ten mice were continuously observed for 14 days to record body weights and death rates. For the other 10 mice, 5 were dissected at 3 and 5 d.p.i. to collect brains, tracheas, lungs, and kidney tissues, as well as to take eye swabs for viral titer determinations.

### 2.9. Virus Titrations

To measure the infectivity of the influenza viruses, 10-fold serial dilutions of the viruses were used to inoculate MDCK cell monolayers at 37 °C for 72 h. After 72 h of infection, the cytopathic effect (CPE) was observed, and the TCID_50_ values were calculated by the Reed and Muench method.

### 2.10. Histopathology Evaluation

Animal necropsies were performed, according to a standard protocol. Samples for histological examination were stored in 10% neutral-buffered formalin (lungs were stored after inflation with formalin), embedded in paraffin, sectioned at 4 μm, and stained with haematoxylin and eosin (H&E) for examination by light microscopy. Pathological sections of the lesion sites were delineated and calculated by Nanozoomer Digital Pathology Image analysis. The lung lesion scores were determined based on alveolar space widening, the presence of inflammatory cells, serous exudate in the alveolar cavity, the infiltration of interstitial inflammatory cells, and vascular dilation and congestion, which were scored from 0 to 4 for each item, according to the degree and area of the lesion. The final score was calculated based on the accumulated scores and reported as the final result.

### 2.11. Quantitative Reverse Transcription-Polymerase Chain Reaction (RT-qPCR)

RNA was extracted from 100 µL of each sample using the RNeasy Mini Kit (Qiagen, Hilden, Germany). A total of 5 µL of RNA was added to a 25 µL reaction for one-step amplification (QuantiTect Probe RT-PCR Kit, Qiagen) and the amplification condition were as follows: 50 °C for 10 min, 95 °C for 10 min, followed by 40 cycles of 95 °C for 15 s, and then 60 °C for 45 s. The primers and probe sequences sets for HA genes of H7N9 viruses: forward primer 5′AGAAATGAAATGGCTCCTGTCAA3′, reverse primer 5′GGTTTTTTCTTGTATTTTTATATGACTTAG3′. The probe sequence was 5′-FAM- AGATAATGCTGCATTCCCGCAGATG -BQH1-3′. In this assay, isolated viral RNA was used as a control.

### 2.12. HI Assays

The serum samples were treated with a receptor-destroying enzyme (RDE; Sigma-Aldrich, St. Louis, MO, USA) at 37 °C for 12-16 h and then incubated at 56 °C for 0.5 h. Next, the treated serum samples were mixed with concentrated cRBCs at 4 °C for 1 h. The supernatants were collected for 10-fold gradient dilutions, and 25 µL of each supernatant was mixed with an equal volume of virus at 4 HA (hemagglutination units) and incubated at room temperature for 40 min. The results were determined using 1% cRBCs.

### 2.13. Nucleotide Sequencing and Analysis

Viral RNA was used for reverse transcription using a high-fidelity PrimeScript™ One Step RT-PCR kit (TaKaRa, Shiga, Japan). The product was sequenced using the dideoxy method on an ABI 3730 DNA sequencer (Applied Biosystems, CA, USA). During the sequencing process, amplification was performed using specific primers. The sequences for this process are available upon request. The sequencing reads obtained were linked using DNAMAN, and the results were compared using the Megalign module in the DNAStar software package.

### 2.14. Statistical Analyses

Differences in animal body weights, viral copy numbers, and viral titers among the different groups were analyzed by one-way analysis of variance (ANOVA) and post-hoc Bonferroni correction analysis. Differences between two groups were analyzed by a Student’s *t*-test using SPSS 11.5 software (IBM SPSS Software Inc., USA). A probability value of <0.05 was considered statistically significant.

## 3. Results

### 3.1. Pathogenicity and Transmissibility of H7N9 Avian Influenza Viruses in Ferrets

#### 3.1.1. Infection Group

After ferrets infected with Th005 or Anhui, the body weight, body temperature, and clinical symptoms were observed, and the virus replication in the upper respiratory tract and tissues were detected.

**Figure 1:**
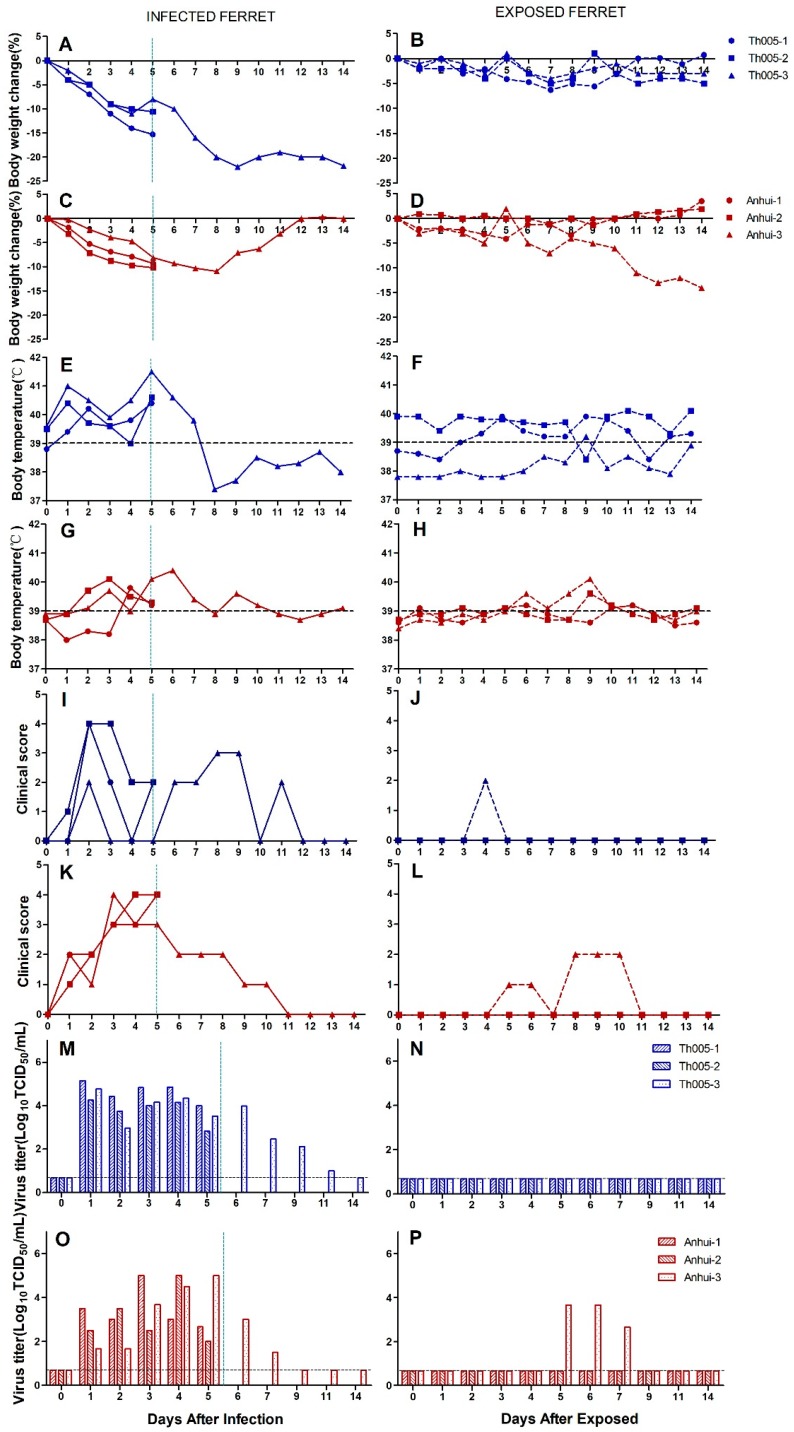

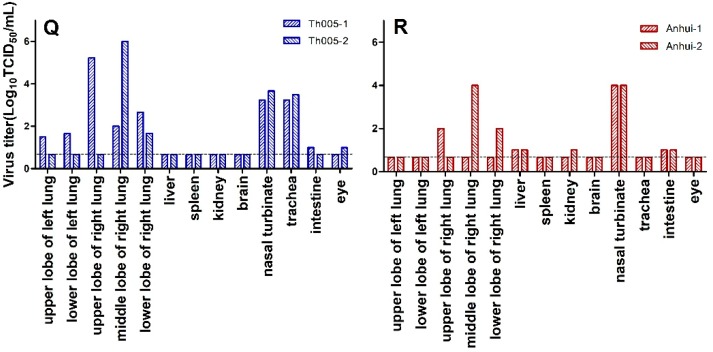
Replication and virulence of A/Guangdong/Th005/2017 (H7N9) and A/Anhui/1/2013 (H7N9) in ferrets and aerosol transmission experiments. Body weight: (**A**) Th005 infection group, (**B**) Th005 transmission group, (**C**) Anhui infection group, and (**D**) Anhui transmission group. Body temperature: (**E**) Th005 infection group, (**F**) Th005 transmission group, (**G**) Anhui infection group, and (**H**) Anhui transmission group. Clinical score: (**I**) Th005 infection group, (**J**) Th005 transmission group, (**K**) Anhui infection group, and (**L**) Anhui transmission group. Viral titers in ferret nasal swabs: (**M**) Th005 infection group, (**N**) Th005 transmission group, (**O**) Anhui infection group, and (**P**) Anhui transmission group. Viral titers in ferret viscera in infection groups: (**Q**) Th005 infection group and (**R**) Anhui infection group. The horizontal dotted lines are the lower limits of detection. The vertical lines indicate that two animals were euthanized and dissected on that day.

**Figure 2:**
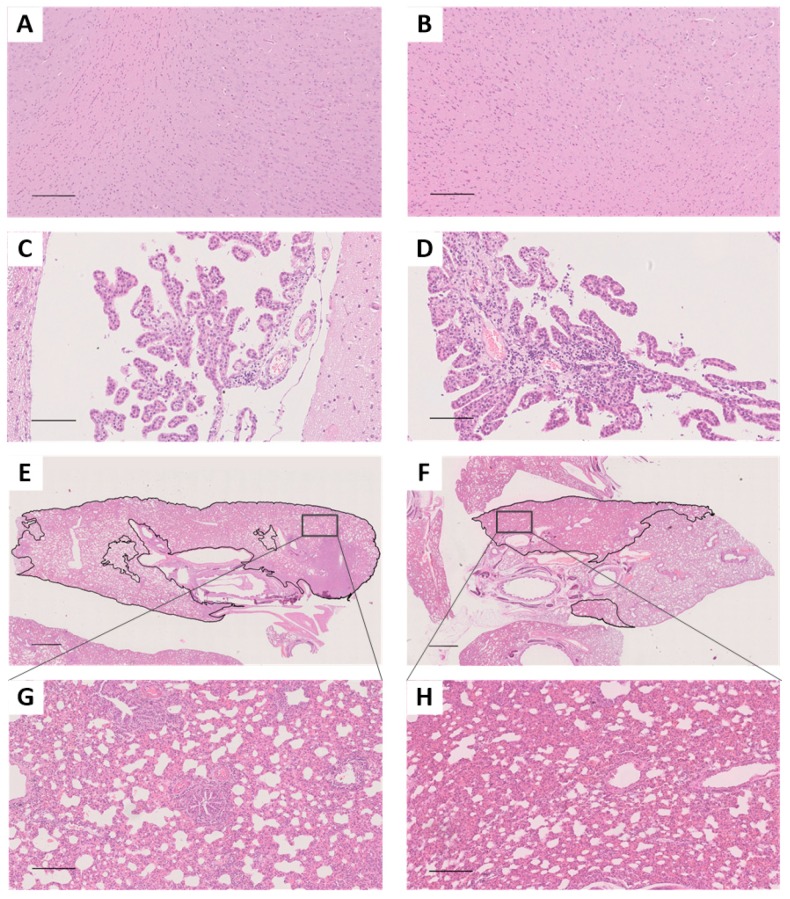
Hematoxylin and eosin staining of brain and lung tissue collected from ferrets at 5 d.p.i. (**A**) Brain parenchyma tissue of the Th005 infection group. (**B**) Brain parenchyma tissue of the Anhui infection group, scale bar is 200 μm. (**C**) Brain choroid plexus tissue of the Th005 infection group. (**D**) Brain choroid plexus tissue of the Anhui infection group, scale bar is 100 μm. (**E**) Lung tissue of the Th005 infection group. (**F**) Lung tissue of the Anhui infection group where the scale bar is 1.5 mm. (**G**) Lung tissue of the Th005 infection group. (**H**) Lung tissue of the Anhui infection group where the scale bar is 200 μm.

All the ferrets in the infection group demonstrated sustained body weight reductions by 1 d.p.i. One of the three ferrets in the Th005 infection group reached its lowest weight at 9 d.p.i., with a 21.8% reduction, and did not return to its pre-infection weight by 14 d.p.i. (Figure 1A). One of the three ferrets in the Anhui infection group reached its lowest point at 8 d.p.i., with a 10.9% reduction. However, this ferret did return to its pre-infection weight by 14 d.p.i. (Figure 1C). The overall body weight reductions of the ferrets were significantly greater in the Th005 infection group than in the Anhui group. In general, the basal temperature of ferrets is 38.5 °C to 40 °C. All ferret temperatures in the Th005 infection group exceeded 40 °C, with one ferret exceeding 41 °C twice during the post-infection monitoring period (Figure 1E). Two of the three ferrets in the Anhui group exceeded 40 °C. However, none of them reached 41 °C at any time during the post-infection monitoring period (Figure 1G). The body temperatures of the ferrets in the Th005 and Anhui infection groups were all significantly increased during the post-infection monitoring period. The highest clinical score among the ferrets in both the Th005 and Anhui infection groups reached a peak value of 4, with symptoms lasting to 12 and 11 d.p.i. in the Th005 and Anhui groups, respectively (Figure 1I,K). Viral shedding in the Th005 and Anhui infection groups continued out to 11 and 7 d.p.i. with peak values of 10^5.15^ and 10^5^ TCID_50_/mL, respectively (Figure 1M,O), which indicates that Th005 viral shedding occurred over a longer period of time. Viral replication could be detected in multiple tissues and organs of infected ferrets from both the Th005 and Anhui infection groups, even though the viral distribution in the tissues was different. Viral replication could be detected in both lobes of the lung in Th005-infected ferrets, compared to Anhui-infected ferrets in which viral replication could be detected in only three-fifths of the lungs. Moreover, the replication titer of Th005 in the lung tissue was 100 times higher than the Anhui strain. Neither virus could be isolated from the brain tissue of ferrets (Figure 1Q,R). Comparing the same location on the same pulmonary lobe after infection, the pulmonary lesion areas in the Th005 group accounted for 91% of the total pulmonary lobe, while the pulmonary lesion areas in the Anhui group accounted for 41% of the total pulmonary lobe. Pathological examination of ferrets infected with Th005 revealed more severe lesions with larger areas than those observed in Anhui-infected ferrets. The mean score of the lung pathological changes in the Th005 group was 9.5, while the mean score in the Anhui group was 7.5. Enlargement of the alveolar septum, inflammatory cell infiltration into the alveolar spaces, vasodilation, and hemorrhages were observed in both groups (Figure 2E–H). No brain tissue lesions were observed in any infected ferrets (Figure 2A–D). Blood samples from the remaining ferrets in the Th005 and Anhui infection groups were collected after 21 days of viral challenge for HI detection and the results were all positive (HI titer of Th005-infected remaining ferret was 1280, HI titer of Anhui-infected remaining ferret was 640).

#### 3.1.2. Transmission Group

The body weight, body temperature, and clinical symptoms of the exposed ferrets were monitored, and the virus replication of the upper respiratory tract of ferrets were continuously detected.

Ferrets in the Th005 transmission group showed no significant reduction in body weight during the observation period (Figure 1B). One of the three ferrets in the Anhui transmission group presented a sustained body weight reduction starting 11 days post-exposure (d.p.e.), reaching its lowest weight at 14 d.p.e., with a 14% reduction (Figure 1D). The body temperatures of the ferrets in the Th005 and Anhui transmission groups as a whole did not exhibit evident changes (Figure 1F and Figure 1H). The clinical scores in the Th005 transmission group accumulated to a value of 2 (Figure 1J) and the clinical scores in the Anhui transmission group accumulated to a value of 8 (Figure 1L). Viral discharge was not detected in the nasal swabs from the ferrets in the Th005 transmission group (Figure 1N), whereas the nasal swab from one ferret in the Anhui transmission group exhibited viral discharge from 5 to 7 d.p.e. (Figure 1P). One ferret in each of the two groups had positive HI results, with the ferret in the Anhui transmission group exhibiting higher anti-H7 antibody levels than the ferret in the Th005 transmission group (HI titre of Th005-exposed ferret was 160, HI titer of Anhui-exposed ferret was 320).

### 3.2. Pathogenicity of H7N9 Avian-Passaged and Mammalian-Passaged Strains in Chickens

#### 3.2.1. The IVPI of Chickens

The Th005 and Anhui strains were intravenously inoculated into six-week-old, white Leghorn chickens. The chickens in the Th005 group all died within 1 d.p.i., with an intravenous pathogenicity index (IVPI) of 3. None of the chickens in the Anhui group exhibited clinical symptoms within 10 d.p.i., with an IVPI of 0. According to the IVPI determination requirements in the OIE document, Anhui is a lowly pathogenic avian influenza virus (LPAIV), while Th005 is a highly pathogenic avian influenza virus (HPAIV).

#### 3.2.2. Pathogenicity in Nasally Inoculated Chickens

After chickens nasal infected with Th005 or Anhui, the body weight, body temperature, survival rate, and clinical symptoms of chickens were observed, and the virus replication in the upper respiratory tract and the cloacal cavity were detected.

**Figure 3:**
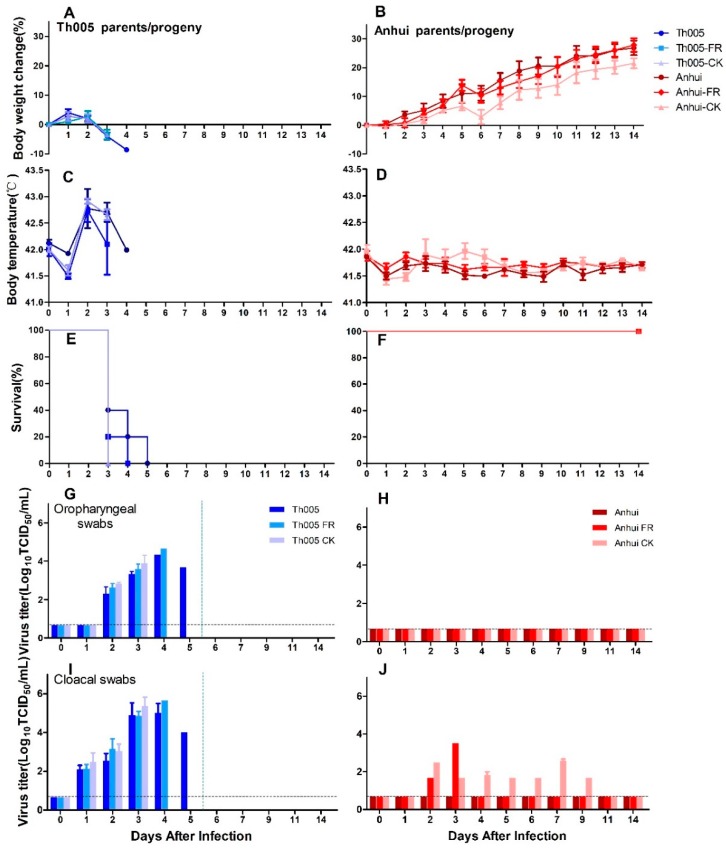
Pathogenicity of Th005, Th005 FR, Th005 CK, Anhui, Anhui FR, and Anhui CK infection in chickens. Body weight change rate: (**A**)Th005, Th005 FR, and Th005 CK. (**B**) Anhui, Anhui FR, and Anhui CK. Body temperature: (**C**)Th005, Th005 FR, and Th005 CK. (**D**)Anhui, Anhui FR, and Anhui CK. Survival rate: (**E**)Th005, Th005 FR, and Th005 CK. (**F**) Anhui, Anhui FR, and Anhui CK. Viral titers in oropharyngeal swabs from chickens: (**G**)Th005, Th005 FR, and Th005 CK. (**H**)Anhui, Anhui FR, and Anhui CK. Viral titers in cloacal swabs from chickens: (**I**) Th005, Th005 FR, and Th005 CK. (**J**)Anhui, Anhui FR, and Anhui CK. The results are displayed as means and SD from duplicates from five independent chickens.

All chickens in the Th005, Th005 FR, and Th005 CK nasal inoculation groups exhibited obvious body weight reductions after infection, with the weights of the chickens continually dropping. Because all the chickens died within three to five days, the monitoring results only lasted until five days after infection (Figure 3A). The chickens in the Anhui, Anhui FR, and Anhui CK groups exhibited sustained body weight increases after infection, with an average daily increase of approximately 2% (Figure 3B). The mean body temperatures of the chickens in the Th005, Th005 FR, and Th005 CK groups exhibited significant increases over basal temperatures, with peak temperatures of over 42 °C at 2 d.p.i. (Figure 3C). The mean body temperatures of the chickens in the Anhui, Anhui FR, and Anhui CK groups fluctuated within the normal range after infection (Figure 3D). Chickens in the Th005, Th005 FR, and Th005 CK groups all died within 5 d.p.i. (Figure 3E). None of the chickens in the Anhui, Anhui FR, and Anhui CK groups died during the 14-day observation period after infection (Figure 3F). At 3 d.p.i., the chickens in the Th005, Th005 FR, and Th005 CK groups exhibited depression, skin surface bruising, cyanosis of the wattles, edema of the face, and death (Figure S1A–D). The Anhui and Anhui FR groups did not exhibit any symptoms, whereas the Anhui CK group exhibited transient edema of the face and conjunctivitis on the fifth day after the viral challenge with symptoms that lasted for one day and then disappeared (Figure S1E,F).

Viruses were detected in the oropharyngeal swabs of chickens in the Th005, Th005 FR, and Th005 CK groups with peak mean values at 3 to 4 d.p.i. of 10^4.33^, 10^4.67^, and 10^3.89^ TCID_50_/mL, respectively. Viral discharge in cloacal swabs from the Th005, Th005 FR, and Th005 CK groups peaked at 3 to 4 d.p.i with values of 10^5^, 10^5.67^, and 10^5.37^ TCID_50_/mL, respectively (Figure 3G,I). Neither the oropharyngeal swabs of chickens infected with Anhui, Anhui FR, and Anhui CK nor the cloacal swabs from the Anhui group exhibited detectable viral titers (Figure 3H). Cloacal swab samples from the chickens in the Anhui FR exhibited viral discharge on 2 and 3 d.p.i. The chickens in the Anhui CK group had detectable viral discharges from 2 to 9 d.p.i. and the viral discharge values in the cloacal swabs peaked at a value of 10^2.58^ TCID_50_/mL on day 7 (Figure 3J).

### 3.3. Pathogenicity of H7N9 Avian-Passaged and Mammalian-Passaged Strains in Mammals

The body weight and survival rate of Th005 or Anhui infected mice were observed, and the virus replication in the tissues were detected.

**Figure 4:**
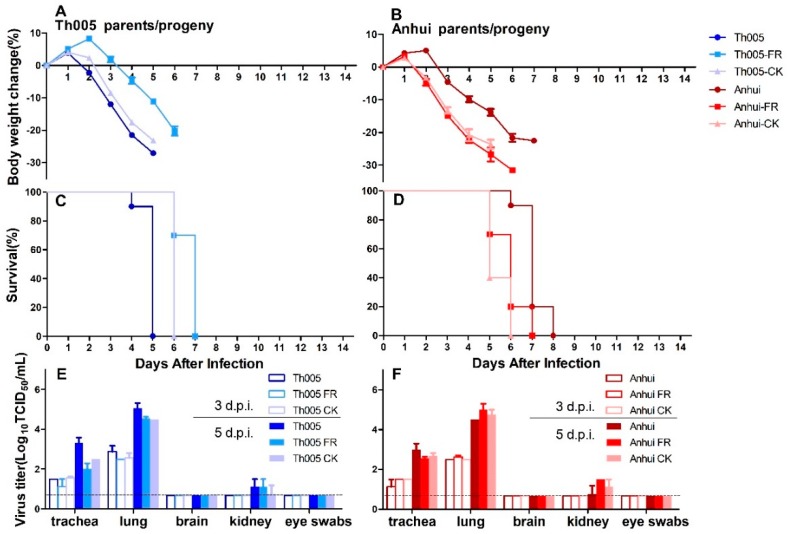
Pathogenicity of Th005, Th005 FR, Th005 CK, Anhui, Anhui FR, and Anhui CK infection in mice. Body weight change rate: (**A**)Th005, Th005 FR, and Th005 CK. (**B**)Anhui, Anhui FR, and Anhui CK. Survival rate: (**C**) Th005, Th005 FR, and Th005 CK. (**D**)Anhui, Anhui FR, and Anhui CK. Viral titers in mice viscera after infection: (**E**) Th005, Th005 FR, and Th005 CK. (**F**) Anhui, Anhui FR, and Anhui CK. The results of A-D are displayed as means and SD from duplicates from ten independent mice, and the results of E, F are displayed as means and SD from duplicates from five independent mice.

The reductions in body weight of the mice in the Th005 group were significantly greater than those in the Anhui group. The reductions in body weight of the mice in the Th005 FR group were less than those in the Th005 group, and the average reductions in the Th005 CK and Th005 groups were not different. The body weights of the mice in the Anhui FR and Anhui CK groups were significantly decreased when compared to those in the Anhui group (Figure 4A,B). The survival time was shorter in the Th005 group than in the Anhui group. The survival time was longer in the Th005 FR and Th005 CK groups when compared to the Th005 group and shorter in the Anhui FR and Anhui CK groups than in the Anhui group (Figure 4C,D). The virus could be detected in the tracheas and lungs of Th005-infected and Anhui-infected mice, as well as in their progeny, with higher viral titers on 5 d.p.i. than on 3 d.p.i. Regardless of whether the samples were taken from the upper or lower respiratory tracts, the mean viral titers in the Th005 group were slightly higher than those in the Th005 FR and Th005 CK groups. Additionally, the Anhui FR and Anhui CK groups exhibited higher viral titers in lungs than did the Anhui group. The virus was barely detectable in the kidney and brain and in eye swabs (Figure 4E,F).

### 3.4. Viral Growth Replication Curves

**Figure 5:**
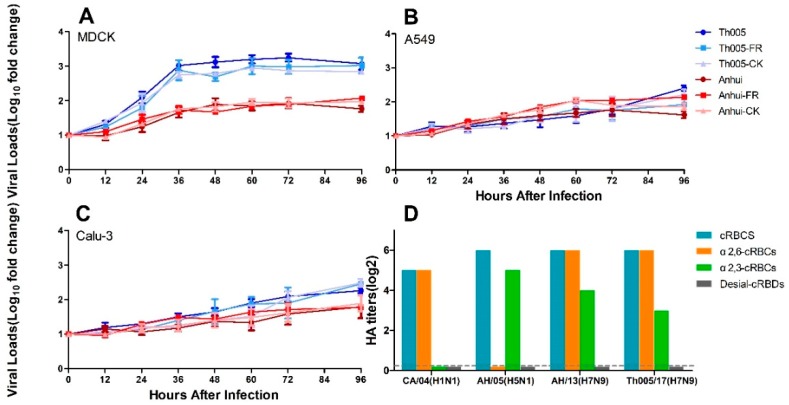
Growth replication curves and viral receptor-binding characteristics of Th005, Th005 FR, Th005 CK, Anhui, Anhui FR, and Anhui CK. (**A**) MDCK cells, (**B**) A549 cells, and (**C**) Calu-3 cells. (**D**) Viral receptor-binding characteristics: cRBCs, untreated chicken red blood cells. α2,6-cRBCs, removal of α2,3 sialic acid protein on the surface of chicken red blood cells by α2,3-sialidase. α2,3-sRBCs, sheep red blood cells containing only α2,3 sialic acid protein. Desialidated cRBCs, VCNA-treated chicken red blood cells with removal of proteins containing α2,3 and α2,6 sialic acid.

The replication rates of the primary viruses and strains passaged in different hosts were not significantly different. In MDCK cells, the RNA copy numbers in the supernatants of the Th005, Th005 FR, and Th005 CK groups were all significantly higher than those observed in the Anhui, Anhui FR, and Anhui CK groups (Figure 5A). Differences in the viral replication curves were also present in Calu-3 cells, but their growth was delayed compared to MDCK cells (Figure 5C). Unlike the viral replication results in the MDCK and Calu-3, Th005, Th005 FR, Th005 CK, Anhui, Anhui FR, and Anhui CK replicated equally poorly in A549 cells (Figure 5B).

### 3.5. Viral Receptor-Binding Characteristics

The sialic acid receptor binding characteristics of the Th005 strain were similar to those of the Anhui strain, which exhibited an affinity for both receptors. The binding affinity of Th005 for the α2,6-sialic acid receptor was equivalent to that of the Anhui strain, which reached 2^6^ HA. The Th005 binding affinity for the α2,3-sialic acid receptor was 2^3^ HA, while the binding affinity of the Anhui strain for the α2,3-sialic acid receptor was 2^4^ HA (Figure 5D). In this study, the CA/07 (H1N1) and AH/05 (H5N1) viruses with selective binding for the receptors were selected to ensure the specificity and effectiveness of the cRBCs used in the agglutination analysis.

### 3.6. Analysis of Mutations in Key Pathogenic Sites (Table 1)

The insertion in the HA cleavage motif was “-KRIA-” of Th005, which was consistent with the principle “-KXXR-” (X = any amino acid and R = arginine) motif observed in highly pathogenic avian influenza viruses [3,13]. The HA-L226Q motif in Th005 was previously reported to be primarily associated with the transition from human-type receptors to avian-type receptors [20]. In this study, only Anhui CK contained N133D/N158-159D double mutations, which would reduce the thermal stability of HA and enhance affinity for the α2,3-sialic acid receptor [16]. According to Yao et al. [24], residues between 362–581 of the PB2 region (V511I, K526R, M535L, N559L, and M570I) can enhance the viral replication capacity in mouse cells. Especially the PB2-R526 motif in this segment might enhance viral transcription and replication in cells and help support the function of PB2-K627 [25].

The NS1-M41K and PB2-R379S regions were excised in the Anhui FR/Anhui CK and Th005 FR strains, respectively. Lys (K) and Arg (R) are basic amino acids, which are usually considered to enhance virulence.

## 4. Discussion

The first human infection with an H7N9 strain appeared in the Yangtze River Delta region in March 2013 and, between January 2013 and January 2019, this virus has caused 1567 infections and 615 deaths [2]. We urgently need to monitor and evaluate novel H7N9 viruses by confirming their pathogenicity and transmissibility in humans and poultry.

As early as 2013, H7N9 strains isolated from patients during the first wave of infection were studied in ferrets for their pathogenicity and transmissibility, as well as the upper respiratory tract symptoms they caused, such as nasal discharge and sneezing. Viral replication was also measured in the trachea, lung, heart, liver, and the olfactory bulb of infected ferrets [27]. The Anhui strain was limited in its airborne transmission between ferrets, with viral replication detected in the exposed ferrets for two to three days. Significant weight loss was observed [10]. Since pigs are the mixing vessel for influenza viruses, we once suspected that H7N9 influenza viruses might tend to infect mammals after adaptive mutation and reassortment during the epidemic. Therefore, the circulation patterns of H7N9 viruses in nature were simulated in pigs to evaluate their variation trends. Studies passaging H7N9 influenza viruses in pigs showed that the avian-derived H7N9 virus (LPAIV) obtained a higher binding capability for α2,6-sialic acid receptors. However, while human-derived H7N9 virus showed no improvement in replication after its passage in pigs, the HA-L226Q substitution appeared, which improved the binding of human-derived H7N9 virus to α2,3-sialic acid receptors [28]. The Th005 strain isolated from the fifth outbreak had slightly increased replication in the upper respiratory tract of ferrets and a significantly longer duration of viral shedding and, additionally, the viral replication titers of Th005 in lung tissues were significantly improved. The presence of the HA-Q226 substitution allowed the virus to effectively bind to the α2,3-sialic acid receptors and improve the viral replication because of the α2,3-sialic acid receptor enrichment [29,30]. This was consistent with our previous predictions about the circulation of the first wave, human-derived H7N9 virus in pigs.

In 2017, we dynamically tracked the changes in the H7N9 virus and evaluated the pathogenicity and transmissibility of several novel human/avian-derived H7N9 viruses [31]. The results showed that avian-derived A/Chicken/Heyuan/16876/2016 (abbreviated 16876)-infected ferrets exhibited a slightly longer disease duration and viral shedding in the upper respiratory tract when compared to the Anhui strain (disease duration = 8.7 and 7 days, viral shedding duration = 8.3 and 5.7 days, respectively). Furthermore, the viral replication in the upper respiratory tracts was 22-fold higher than that for the Anhui strain. Thus, the 16876 strain had a higher pathogenicity compared to that of the Anhui strain. In addition, the pathogenicity of the A/Shenzhen/Th001/2016 (LPAIV), A/Guangdong/Th008/2017 (HPAIV), and A/Chicken/Huizhou/HZ-3/2017 (HPAIV) strains in ferrets was lower than that of the Anhui strain. Although the 16876 strain had the highest α2,3-sialic acid receptor binding affinity, it did not exhibit effective airborne transmission between ferrets. Tracing the above strains is beneficial in understanding the dynamic alterations in novel H7N9 viruses, and we are committed to tracking the variation trends of these viruses in nature to predict future trends. The viruses were not genetically modified, and, thus, these data can provide a scientific basis for the risk assessment of novel H7N9 viruses. Dynamic evaluations in the same laboratory are beneficial to eliminate errors in assessment results from different studies.

A study evaluated the human-derived A/Guangdong/17SF003/2016 (HPAIV, abbreviated 17SF003) strain and observed that one-quarter of infected ferrets succumbed to infection, with significantly increased replication titers in lung tissues that were 280-fold higher than those with the Anhui strain [32]. In addition, viral replication was detected in the brain tissue of ferrets infected with the 17SF003 strain. We investigated the pathogenicity of the A/Guangdong/Th008/2017 (abbreviated Th008) strain in ferrets (Ke et al. [8] reported that Th008 and 17SF003 are the same stain of virus). Although we did not detect the same organ distributions of the Th008 strain in infected ferrets, the viral replication in the upper respiratory tract was not higher than with the Anhui strain. We speculated that the reason for the varied pathogenicity of Th008-infected ferrets might be because of the challenge dose or the mutations that occurred in the Th008 strain during passage in different laboratories (mutations might not have occurred in key sites, and this situation might be caused by genetic polymorphisms). Considering that we do not know the specific cause of death in the ferrets infected with 17SF003, the specific genetic differences between the 17SF003 and Th008 strains are worthy of further investigation. Another ferret study confirmed that the avian-derived A/Chicken/Guangdong/SD008/2017 (HPAIV, abbreviated SD008) strain could be detected in the brains and throughout the lungs [13]. Neither the human-derived 17SF003 strain nor the avian-derived SD008 strain can efficiently transmit via respiratory droplets.

In our study, we simulated the natural route of infection and found that Th005 could replicate efficiently in the upper and lower respiratory tracts of ferrets. Compared with the Anhui strain, the rate of weight loss in the Th005-infected ferrets was up to 21.8% (the peak weight loss of Anhui-infected ferrets was 10.9%). The peak viral replication titers in the lungs of Th005-infected ferrets were 100-fold higher than those of Anhui-infected ferrets, and the viral shedding period lasted for 11 days. The above results indicated that the pathogenicity of the Th005 strain in mammals increased. Compared to patients in the first wave of the epidemic, patients in the fifth wave were found to have more acute illnesses, and the case fatality rate increased from 30% to 40% [5,6,7,8,9]. Neurological symptoms were not observed and the virus was unable to be isolated from the brain tissue after Th005 infection in humans (patients in which the virus could be isolated), ferrets, or mice, which suggests that the neurovirulence might vary by strains. In addition, similar to the Anhui strain, the Th005 strain had limited airborne transmission between ferrets. The amino acid at HA-186 of Th005 was valine (Q), with the addition of HA-I48V, R57K, and S128N substitutions, which contributed to α2,6-sialic acid receptor binding [15,17,33]. The molecular basis for the high pathogenicity of Th005 in poultry might come from the multi-basic cleavage site in HA [13,14]. In addition, HA-Q226 enhanced the avian receptor affinity of the virus [33]. The data in vitro also confirmed that H7N9 replicated better in MDCK cells, which possess high expression of both α-2,6-sialic and α-2,3-sialic acid receptors. The α2,6-sialic acid receptor content in Calu-3 cells was three-fold higher than that of the α2,3-sialic acid receptor, while the content of both sialic acid receptors was relatively low in A549 cells [34,35]. In the above cells, the replication of Th005 decreased. In the lower respiratory tract of humans and ferrets, α2,3-sialic and α2,6-sialic acid receptors are mixed [36]. The replication titres of Th005 in the ferret lung tissue were 100-fold higher than those of the Anhui strain, which also proved that there is higher replication in the lower respiratory tract. Previous research had confirmed that the PB2-K526R substitution of the A/Chicken/Zhejiang/DTIDZJU01/2013 strain can coordinate with PB2-K627 to increase viral replication [25]. We speculated that the PB2-R526/K627 site in Th005 (PB2-K526/K627 in Anhui) might be the molecular basis for the higher replication of this virus in different cells and animal organs when compared to the Anhui strain.

To observe the possible changes in wild strains after circulation in natural hosts, we simulated the circulation of viruses in nature and analyzed the pathogenic changes of these viruses in mice and avians after passage in ferrets and chickens. It turned out that the lethality of Th005 in mice did not change after passage in ferrets or chickens. Similarly, Th005 maintained high lethality in chickens after passage in both ferrets and chickens. This suggested that Th005 could not only infect both mammals and poultry but also maintain high replication after circulation among them, which is very alarming. In addition, the IVPI of Th005 was confirmed to be 3, with all the chickens dying within one day of infection (the IVPI of SD008 was 3, which exhibited pathogenicity that was no higher than that of Anhui [13]). Although the human-derived Anhui strain did not induce any symptoms after infection of chickens, cloacal virus shedding was continuously detected after passage in mammals or birds (two days for the Anhui FR versus eight days for the Anhui CK). After circulating in different hosts, the Anhui strain was continuously discharged into nature, which gives H7N9 the opportunity to come into contact with other hosts and enhances the chances of a “host-jump.” Our previous results with pig-passaged H7N9 indicated that the human-derived H7N9 virus might obtain an HA-L226Q substitution in pigs to improve its affinity for the avian receptor. The results supported the prediction that avian-derived H7N9 might enhance its ability to bind to human receptors during the passage in pigs, which means that, regardless of the viral source, these viruses will be able to replicate both in mammals and poultry after circulation. Our current results also showed that cloacal Th005 viral shedding titers in chickens could reach 1.74E5 ± 2.01E5 and could lead to rapidly fatal outcomes in sick chickens. This demonstrated a cumulative effect, in which the H7N9 viruses were able to come into contact with poultry and wild birds in a very insidious way and circulate through aquaculture to live poultry markets and wild bird migration routes, which poses a potential risk to any poultry, wild birds, or humans related to this transmission chain. As for the function-related mutants, compared with Anhui, there were three amino acid substitutions in the HA, PA, and NS1 segments of Anhui FR, and 10 amino acid substitutions in the HA, PB2, PA, and NS1 segment of Anhui CK. These were consistent with the accumulated virulence of H7N9 viruses circulated in different hosts. Compared with Th005, only one mutation at the site 379 in PB2 of Th005 FR. The above suggested that H7N9 viruses isolated in 2013 can be adapted further and accumulate virulence in mammals and avians, whereas H7N9 viruses isolated in 2017 had already adapted to mammals and avians. It is reasonable to assume that the fifth wave of this outbreak in humans was not due to effective human-to-human transmission, but, rather, the improvements to pathogenicity in poultry and the expanded viral distribution after reassortment in this hidden circulation cycle. To prevent an epidemic and further mutations of the pathogens in this circulation chain, control of poultry farms, live poultry markets, and the overlapping areas between migrating wild birds and humans should be strengthened.

## Figures and Tables

**Table 1:** Comparison of viral amino acids of Th005, Th005 FR, Th005 CK, Anhui, Anhui FR, and Anhui CK.

Protein	Amino Acid No.	Anhui	Anhui FR	Anhui CK	Th005	Th005 FR	Th005 CK	Function		Reference(s)
HA	Cleavage	——	——	——	KRIA	KRIA	KRIA	Avian pathogenicity		[13,14]
	48	I	I	I	V	V	V	Increase human-type receptor affinity		[15]
	57	R	R	R	K	K	K			
	122	A	A	A	P	P	P	Influence human immune response		[16]
	128	S	S	S	N	N	N	Increase human-type receptor affinity		[17]
	135	A	A	A	V	V	V	Modulate receptor affinity		[18]
	133	N	N	D	N	N	N	Increase avian-type receptor affinity		[16,19]
	158-159	D	N	D	N	N	N			
	226	L	L	L	Q	Q	Q	Alter receptor specificity		[20]
NA	145	G	G	G	E	E	E	Glycosylation site		[21]
	357	A	A	A	D	D	D	Influence neighboring NA tetramers		
PB1	525	V	V	V	I	I	I	Influence replication in varied species		[22]
PB2	191	K	K	K	E	E	E	Influence polymerase activity		[23]
	292	V	V	V	I	I	I	Influence replication in human		[22]
	379	R	R	R	R	S	R		Influence replication in mouse cells	[24,25,26]
	382	I	I	N	I	I	I			
	383	Q	Q	S	Q	Q	Q			
	511	V	V	I	I	I	I			
	526	K	K	R	R	R	R	Enhance the 627K and 701N function		
	535	M	M	L	L	L	L	Restore the polymerase activity		
	559	N	N	T	T	T	T	Influence polymerase activity		
	570	M	M	I	I	I	I			
	647	I	I	I	M	M	M	Contribute to the phenotype		[22,24]
PA	55	D	D	K	D	D	D	Host signature amino acids		[26]
	409	N	S	N	N	N	N			
NS1	41	M	K	K	K	K	K	Not Clear		[22]
	124	M	M	M	V	V	V			
M2	24	E	E	E	D	D	D	Not Clear		

The H3 and N2 numbering system was used. “——”represented deletion of a multi-basic cleavage site in HA.

## Supplementary Materials

The following are available online at https://www.mdpi.com/1999-4915/11/6/494/s1. **Figure S1.** Clinical symptoms of chickens infected via the nasal route.
