# Supplementary material for: Human-Derived A/Guangdong/Th005/2017 (H7N9) Exhibits Extremely High Replication in the Lungs of Ferrets and Is Highly Pathogenic in Chickens
Source: Viruses. 2019 May 29;11(6):494. doi: 10.3390/v11060494 (PMC6630577; doi:10.3390/v11060494)
Supplement: Supplementary file 1 [file viruses-11-00494-s001.pdf]

Supplementary Materials

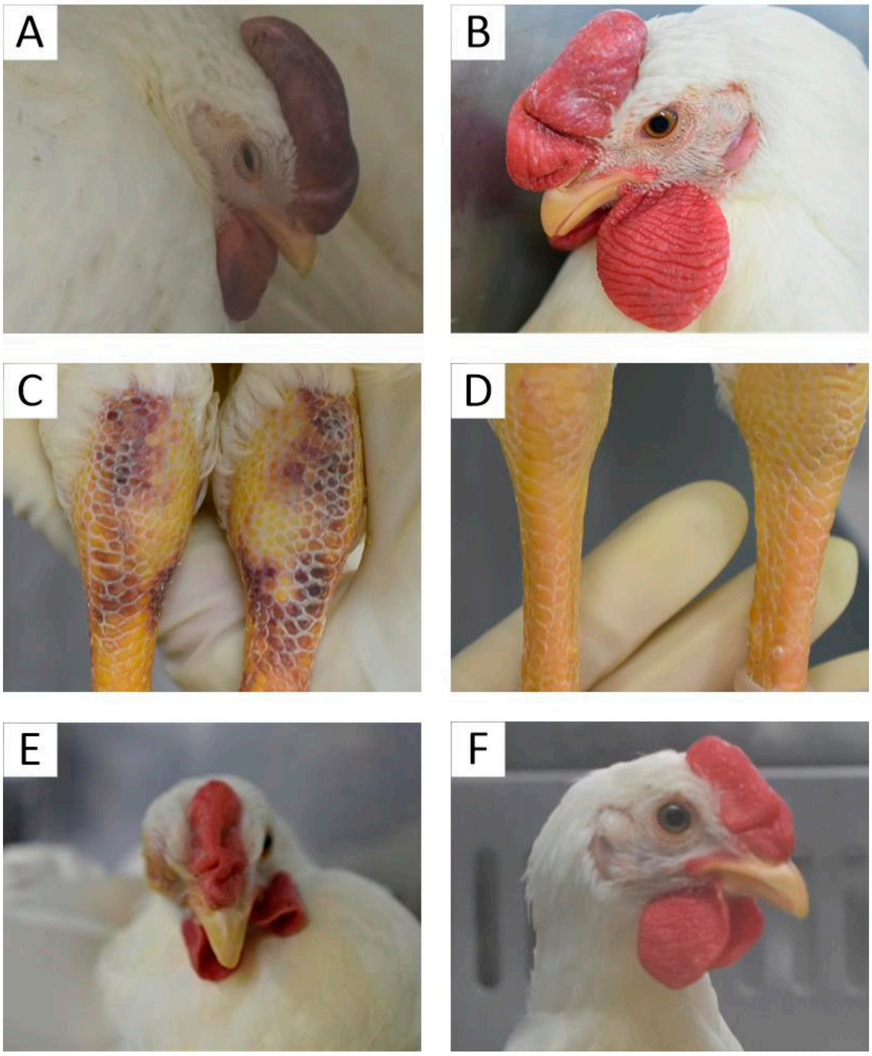

Figure S1 Clinical symptoms of chickens infected via the nasal route. (**A**)Clinical symptoms of chickens after infection with Th005 and its passaged strains: depression, cyanosis of wattles, and edema of the face. (**B**)Chicken without infection. (**C**)Skin surface bruising in chicken legs after infected with Th005 and its passaged strains. (**D**)Chicken legs without infection. (**E**) Conjunctivitis and edema of the face in an Anhui CK-infected chicken. (**F**) Conjunctivitis and edema of the face in the Anhui CK-infected chicken disappeared next day.
